# Chemiluminescence Detection in the Study of Free-Radical Reactions. Part 2. Luminescent Additives That Increase the Chemiluminescence Quantum Yield

**DOI:** 10.32607/actanaturae.11427

**Published:** 2022

**Authors:** L. A. Romodin

**Affiliations:** The A. I. Burnazyan Federal Medical Biophysical Center of the Federal Medical Biological Agency of Russia, Moscow, 123098 Russia

**Keywords:** free radical reactions, apoptosis, ferroptosis, chemiluminescence, lipid peroxidation, reactive oxygen species, chemiluminescence enhancers, coumarin derivatives

## Abstract

The present review examines the use of chemiluminescence detection to evaluate the course of free radical reactions in biological model systems. The application of the method is analyzed by using luminescent additives that enhance the luminescence thanks to a triplet–singlet transfer of the electron excitation energy from radical reaction products and its emission in the form of light with a high quantum yield; these additives are called chemiluminescence enhancers or activators. Examples of these substances are provided; differences between the so-called chemical and physical enhancers are described; coumarin derivatives, as the most promising chemiluminescence enhancers for studying lipid peroxidation, are considered in detail. The main problems related to the use of coumarin derivatives are defined, and possible ways of solving these problems are presented. Intrinsic chemiluminescence and the mechanism of luminescence accompanying biomolecule peroxidation are discussed in the first part of the review.

## INTRODUCTION

Due to the extremely low intensity of intrinsic chemiluminescence, the mechanisms of which are described in the first part of the review [1], it is quite difficult to detect. In addition, it is often necessary to study reactions that include the formation and participation of specific radicals such as lipid peroxidation processes; i.e., to evaluate the presence of lipid radicals in the system under study. However, the method used to detect intrinsic chemiluminescence is nonspecific.

In order to increase the chemiluminescence intensity, specific substances that enhance it are added to the system. These substances are called chemiluminescence enhancers or activators. A subgroup of these substances is called chemiluminescent probes. However, this term is often used randomly. From the chemical point of view, the correct terms would be a chemiluminescent reagent and luminescent additive. The ambiguity of the term activator has to do with the fact that it is generally interpreted as the ability of a particular compound to interact chemically, while the specific meaning of the word is the active part of a concentration. The monograph [2] presents a short list of terms related to the topic of chemiluminescence. This list contains the term initiator, which is considered "a chemically active substance that creates primary active centers and thereby increases the rate of the reaction that provides active products and changes the quantum yield of excitation." The term activator may also fall under this definition. It should be noted that luminescent additives in biological systems come in aqueous solution with a pH of ~7, where they can exhibit low solubility. leading to their aggregation. The interaction of phagocytes with additive microparticles activates the production of reactive oxygen species (ROS) [3, 4]; thus, the term activator can be used in this system in relation to the additives under discussion.

The term activator can be used when describing systems with chemically initiated electron-exchange luminescence: e.g., chemiluminescence of oxalate esters [5]. Introduction of a fluorophore with a low ionization potential to the system leads to electron transfer from this compound to the intermediate. This is followed by reverse electron transfer, leading to fluorophore excitation, which then becomes a chemiluminescence emitter. However, a luminescent additive is most often called an activator. The definition of the latter in that case is a compound that has a high quantum yield of emission and enhances luminescence owing to physical migration of the energy of the electronically excited state (EES) without a change in the excitation quantum yields of radical reaction products and the reaction speed [2, 6, 7].

The increase in luminescence in the presence of these substances is the result of electronic excitation energy (EEE) migration from the reaction products to the additive, which (or its product of interaction with the radical reaction product, i.e. the excitation donor) is a more efficient light emitter than the excited donor compound. In 1963, R.F. Vasil’ev studied the mechanism of chemiluminescence enhancement upon addition of anthracene derivatives to the ketone products of free radical oxidation of hydrocarbon substrates in the triplet EES [8]. The resulting excited molecules of anthracene derivatives were not in the triplet but singlet EES. Thus, a fundamental photophysical process that is widely used to enhance luminescence in chemiluminescent systems, namely physical enhancement of chemiluminescence as a result of a triplet–singlet energy transfer in the liquid phase, was studied in detail [8]. It should be noted that chemiluminescence enhancement in the presence of anthracene derivatives had been demonstrated a year earlier [9]. However, the enhancement mechanism had not been elucidated, yet. An analysis of the action of anthracene and its derivatives showed that anthracene itself is less effective than its halogenated derivatives: in particular 9,10-dibromoanthracene [9, 10, 11]. The corresponding value of the exclusion coefficient of the triplet–singlet transition, which is calculated as the ratio of the reaction rate constant to the diffusion rate constant, is 10^-2^ [11].

Chemiluminescence enhancement can be schematically represented as follows:

P^* →*k*3^ P+hv (non-activated chemiluminescence with a quantum yield of Qlum1).

P^*^ + enhancer (activator) → P + enhancer^* → *k*3enh → *k*3enh^ P + enhancer + hv.

This is activated chemiluminescence with a quantum yield of Q_lum2_. Note that Q_lum1_ < < Q_lum2_.

An important chemiluminescence enhancer characteristic is not only the chemiluminescence quantum yield value, but also the same value multiplied by the molar extinction coefficient of the given compound, since this multiplication is directly proportional to the luminescence intensity [12].

R.F. Vassil’ev and V.A. Belyakov provided the basis for our understanding of the triplet–triplet and triplet–singlet EES energy transfer for the quantitative study of chemiluminescent reactions [11]. In particular, the relationship between the rates of EEE migration from the radical reaction product (EEE donor), EEE acceptor (chemiluminescence enhancer) concentration (let us denote it by A), and chemiluminescence intensity in the absence (J_0_) and presence (J) of the excitation acceptor has been determined:

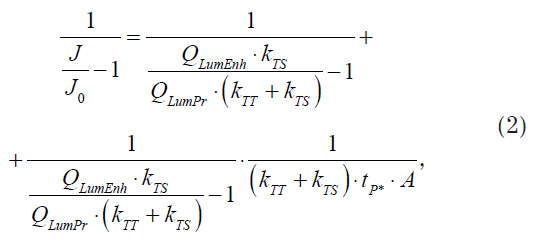



where Q_LumEnh_ is the quantum yield of the luminescence enhancer (EEE acceptor), Q_LumPr_ is the quantum yield of the excited product of the radical reaction (EEE donor), t_P*_ – average donor excitation lifetime in the absence of EEE acceptor, k_TT_ is the rate constant of the triplet–triplet EEE transfer (chemiluminescence quenching), and k_TS_ is the rate constant of the triplet–singlet EEE transfer to the acceptor molecule. The rate constant of the triplet–triplet transfer, which does not result in luminescence, is higher than that of the triplet–singlet transfer [11]. The non-emissive triplet–triplet energy transfer is more pronounced in 1,2-dioxetanone decomposition than in the case of 1,2-dioxetane, which determines the lower emission efficiency of the activated decomposition of dioxetanone compared to that of dioxetane [13].

However, different chemiluminescence enhancers have different mechanisms of receiving the EEE from the radical reaction products. There are two groups of chemiluminescence enhancers. There is some ambiguity in their terminology that should be mentioned. Luminescent additives of the first group react chemically with the participants and products of a free radical reaction and result in the EES, with a quantum yield much higher than that of intrinsic chemiluminescence. According to the terminology proposed by A.I. Zhuravlyov [2], these substances are called chemiluminescent probes. Yu.A. Vladimirov calls these substances chemical activators of chemiluminescence [6]. From the chemical point of view, a chemiluminescent reagent would be a better term for these substances, since they substitute the reaction pathways of ROS, resulting in ultra-weak chemiluminescence under natural conditions, with other pathways leading to higher chemiluminescence. Substances of the second group of luminescent additives generate the EES without interacting chemically with the system components. Representatives of the Yu.A. Vladimirov scientific school [6, 14, 15, 16] call these substances physical activators of chemiluminescence, thus extending the term activator to both groups of chemiluminescent reagents. The authors of [2] use the term activator to designate physical chemiluminescence activators only.

However, it is important to note that the above classification is largely theoretical: most luminescent additives cannot be clearly assigned to a specific group. This is because the chemiluminescence mechanism for most of them is not fully understood. The simple fact of an increase in the intensity of detected chemiluminescence in response to introduction of an additive does not allow one to classify this additive as either a chemical or physical activator.

We should mention that chemiluminescence enhancers were divided into two groups in one of the first studies involving them [10]. Activators were characterized as either bad activators, those without chemical stability and capable of quenching luminescence at high concentrations, or good activators, those with chemical stability and a chemiluminescence enhancement coefficient that increases monotonically with an increase in concentration (see the formula for calculating the luminescence enhancement coefficient in [10]).

## EXAMPLES OF SUBSTANCES THAT ENHANCE CHEMILUMINESCENCE

The phenomenon of chemiluminescence enhancement was first observed upon using anthracene derivatives [8, 9, 10]. Later, dibromoanthracene, which is a physical chemiluminescence enhancer, was used to study the decomposition of polymers during their oxidation by a peroxide compound [17]; dibromoanthracene and 9,10-diphenylanthracene were utilized to explore the chemiluminescence of a ascorbate- and hemoglobin-dependent brain [18]. Anthracene was used to study dioxetane and dioxetanone decomposition accompanied by EES generation [13].

Luminol (5-amino-2,3-dihydro-1,4-phthalazinedione) is the most common chemiluminescent reagent [19, 20, 21, 22, 23, 24, 25, 26, 27, 28]. In the first half of the 20th century, luminol was known as a substance that could generate chemiluminescence upon oxidation [29]. Luminol was first used as a chemiluminescence activator in the biological system by R.C. Allen et al. when studying the immune response of polymorphonuclear leukocytes in 1972 [30].

The mechanism of luminescence generated by luminol oxidation involves the formation of 4-hydroperoxy-1-oxy-5-aminophthalazin-4-olate, a hydroperoxide product of luminol interaction with ROS [31], chloramines in the case of hydrogen peroxide [32], and oxidized peroxidase forms at certain stages of the peroxidase catalytic cycle [6]. This compound is then naturally converted to 2,3-peroxy-di[hydroxymethyleneyl]phenylamine containing an endoperoxide moiety that is eventually cleaved to form a EES hydroaminophthalate ion. This ion emits a photon when returning to its ground state (the mechanism of luminol interaction with various substances is described in detail in [6, 31, 33, 34]). Aside from luminol, isoluminol, which activates luminescence through a similar mechanism, is sometimes used [35, 36, 37].

Luminol is utilized to evaluate total antioxidant activity based on its reaction with 2,2’-azobis(2-amidinopropane) [38, 39] and in various chemiluminescent methods for hydrogen peroxide detection (see review [40]). Some techniques use several substances as chemiluminescent reagents at once. For instance, addition of fluorescein to the system increases the chemiluminescence intensity in the presence of luminol [41]. An increase in luminescence intensity upon addition of some phenols to the horseradish peroxidase–H_2_O_2_– luminol system was also reported [42]. At the same time, so-called non-enhancer phenols inhibit chemiluminescence in the horseradish peroxidase–H_2_O_2_– luminol–4-iodophenol system [43]. These phenols, except for 4-iodophenol, compete with each other as luminol substrates. Luminol remains the most often used substance to determine the immune reactivity of leukocytes [37, 44, 45]; it is also utilized to study lipid peroxidase reactions [24]. The widespread use of luminol is due to the high quantum yield of its luminescence. However, the chemiluminescence enhanced by luminol is nonspecific. Therefore, it is impossible to determine exactly what free radical reactions – and in what proportions – take place in the sample when using laminol.

There are even more specific chemiluminescent reagents, such as the luciferin–luciferase system [46] (luciferase can also have other substrates bedsides luciferin [46]). It is utilized to detect ATP molecules [47]. This system can be also used to solve a large number of other tasks.

Another specific chemical chemiluminescent reagent is coelenterazine (2-(4-hydroxybenzyl)-6-(4-hydrophenol)-8-benzyl-3,7-dihydroimidazo[1,2-alpha] pyrazine-3-one), which is used to evaluate the level of the superoxide radical O_2_·–.

Lucigenin is one of the most frequently used reagents to detect the superoxide radical [6, 48]. It can also be applied to the study of xanthine and hypoxanthine oxidation by xanthine oxidase [49] to detect the superoxide radical formed as the result of NADPH oxidase activity [49, 50, 51, 52] and in ether mitochondria of intact cells [53] or an isolated mitochondrial suspension [54, 55]. Lucigenin-based techniques have recently been developed to detect dopamine [56] and glutathione [57]. In both cases, lucigenin was part of a relatively complex test system (the hypothetical mechanisms of lucigenin-dependent chemiluminescence activation in various systems are discussed in detail in the review [6]).

Fluorescein, which has a high quantum yield of the triplet state [58], is also utilized as a chemiluminescent reagent in one of the hydrogen peroxide detection-based methods [40].

Deamination of amino acids during their oxidation by H_2_O_2_ in the presence of Fe^2+^ ions was studied with the use of ethidium bromide as a chemiluminescent reagent [59]. An increase in the ethidium bromide concentration up to 100 μM in the system under study was shown to be accompanied by a growth in luminescence intensity and its further drop at higher concentrations of ethidium bromide. Furthermore, 1 mM ethidium bromide significantly inhibited amino acid oxidation.

Despite the fact that chemiluminescent probes often cause a greater increase in luminescence, since they are directly involved in the processes occurring in the system under study, they are not suitable for fundamental research, including the study of lipid peroxidation processes. Physical enhancers of chemiluminescence that increase the luminescence quantum yield owing to the resonance transfer of the EEE of reaction products without chemically interacting with the reaction participants and products should be used in that case [60, 61, 62]. This approach is fully consistent with the principle of non-interference with the system under study.

**Fig. 1:**
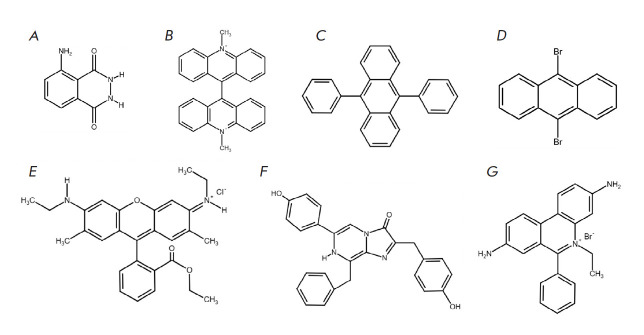
Structural formulas of the substances used as chemical enhancers (activators) of chemiluminescence: luminol (*A*), lucigenin (*B*), 9,10-diphenylanthracene (*C*), 9,10-dibromanthracene (*D*), rhodamine 6G (*E*), coelenterazine (*F*), and ethidium bromide (*G*)

Figure 1 presents the formulas of some of the substances used as luminescent reagents in a number of studies.

## SEARCH FOR PHYSICAL ENHANCERS OF THE CHEMILUMINESCENCE ACCOMPANYING LIPID PEROXIDATION

The interaction of a chemiluminescent probe with components of the system under study presents a serious problem when using these probes in fundamental research. This is because the analyzed chemiluminescent signal is received not from the lipid substrate–peroxidase–hydrogen peroxide system but from the lipid substrate–peroxidase–hydrogen peroxide–chemiluminescence activator system. These data cannot be considered completely adequate for application to living organisms.

An important contribution to our understanding of the chemiluminescence enhancers used in free radical reactions involving lipids was made by V.S. Sharov. In the 1980s, the possibility of using various lanthanides to enhance chemiluminescence was studied. It was suggested that this process is based on intermolecular energy transfer from the products formed in free radical reactions of peroxides to the 4f shell of the lanthanide ion [63]. An example is the data presented in [64]; this led to the conclusion that Tb3+ ions can be used as a physical enhancer of chemiluminescence to study lipid peroxidation reactions. Before that, europium complexed with tetracycline was shown to increase chemiluminescence intensity in lipid peroxidation [65]. However, lanthanide ions are not suitable for research in biological systems due to the following reasons. Chemiluminescence quenching was discovered as early as in the 1980s when using lanthanide ions in biological model systems. This was explained by the fact that lanthanide ions can easily form complexes with the buffer components, which often leads to the loss of their ability to enhance chemiluminescence [65].

In addition, the study of the mechanism of chemiluminescence enhanced by Eu3+ ions complexed with 2,2-dimethyl-6,6,7,7,8,8,8-heptafluoro-3,5-octanedione in the presence of dimethyldioxirane (a model organic peroxide) showed that the lanthanide complex reacts chemically with this organic peroxide. The NMR analysis of the resulting mixture and the photophysical characteristics of the isolated reaction product differed from those of the initial europium chelate. Similar results were also obtained for Eu3+ ion complexed with 2-thenoyltrifluoroacetone, 2,2,6,6-tetramethyl-3,5-heptanedione (dipivaloylmethane), and tris[3-(trifluoromethylhydroxymethylene)d-camphorate]; in the case of the complex with the latter compound in the excess of dimethyldioxirane, chemiluminescence not characteristic of the Eu^3+^ ion but due to an unknown emitter was observed [66].

Apparently, the chemiluminescence of lanthanide chelates can be a result of their interaction with organic peroxides [67]. This conclusion is supported by the assumption that the dioxirane intermediate plays a key role in chemiluminescence generation in the solid-phase reaction between potassium peroxymonosulfate and europium nitrate hexahydrate in the presence of acetone vapor, although the Eu^3+^ ion is the direct emitter [66, 67]. It should also be noted that the Nd^3+^ and Yb^3+^ ions act as chemiluminescence activators, similarly to Eu^3+^ ions in the decomposition of organic peroxides [66].

However, it is important to add that it is the complex of lanthanide ions, but not the ions emitting photons by receiving the EEE from the chelating agent, that is called the chemical activator [66, 67].

Therefore, when searching for an optimal chemiluminescence enhancer, it is necessary to use substances that can undergo triplet–singlet transitions with a high degree of probability. This is due to the fact that the products formed in the disproportionation of lipid peroxide radicals are in the triplet EES [11]. Despite the indicated disadvantages, the above-mentioned lanthanide complexes have the required characteristic. This requirement is also met by low-molecular-weight organic substances containing conjugated cyclic groups. An example is the histological dye Nile blue, which is used as an enhancer of chemiluminescence accompanying Fe^2+^-induced oxidation of lipids [68].

Rhodamine 6G, a xanthene family substance, was used as a physical chemiluminescence activator with a high quantum yield to study tetraoxane decomposition by Fe^2+^ inorganic salts (the comparison of the kinetic dependences of the activated and intrinsic chemiluminescence for the system is presented as evidence) [69]. Coumarin derivatives have similar properties. Such quinolizidine derivatives of coumarin as coumarin-314 (C-314), coumarin-334 (C-334), and coumarin-525 (C-525) act as chemiluminescence enhancers in lipid peroxidation reactions [16, 60, 61, 62, 70]. Because of the selective chemiluminescence enhancement caused by free radical reactions involving lipids, these substances are most suitable for studying lipid peroxidation processes.

**Coumarin derivatives and their use in chemiluminescence detection**

Coumarins are a group of organic compounds that includes unsaturated aromatic lactones: 5,6-benzo-α- pyrone (cis-ortho-hydroxycinnamic acid lactone) derivatives (coumarin or 5,6-benzo-pyran-2-one) [71]. Many members of this group are used as laser dyes [72]. Coumarin derivatives with a substitution at the 7^th^ position (7-hydroxy-4-methylcoumarin and 7-amino-4-methylcoumarin are provided as an example) are effective fluorophores that emit in the visible region [12].

**Fig. 2:**
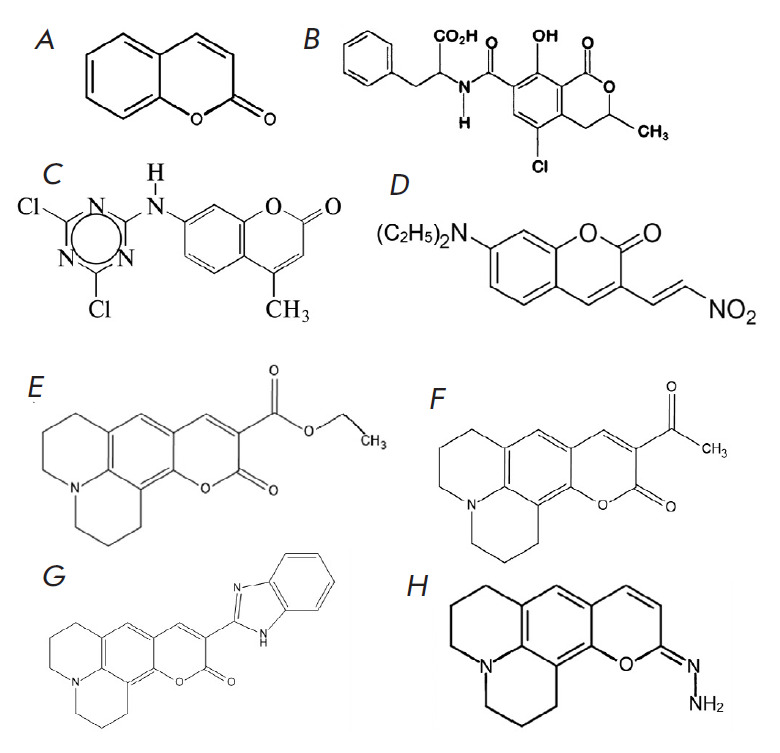
Coumarin (*A*) and its derivatives: ochratoxin A (*B*), DTMC (*C*), 3-(2-nitrovinyl), 7-(diethylamino)coumarin (*D*), C-314 (*E*), C-334 (*F*), C-525 (*G*), and PFM4 (*H*)

Studies using coumarin derivatives as indicators or part of an indicator system deserve special attention. The structural formulas of the coumarin derivatives used as chemiluminescence enhancers are shown in Fig. 2. The coumarin derivative obtained by condensing nitromethane with coumarinyl aldehyde can selectively detect specific cyanide anions [73]. Nucleophilic aromatic substitution of hydrogen with cyanide in the coumarin molecule changes its color and increases the fluorescence intensity (excitation wavelength 365 nm) to an extent that the fluorescence can be observed even with the naked eye. The detection limit is < 3 μM cyanide (dissolved in a acetonitrile medium): the coumarin group generates a bright blue fluorescent signal. The substances 6,7-dihydroxy-4-methyl-8-formylcoumarin and 3,4-benzo-7-hydroxy-8-formylcoumarin can also be used as chromogenic and fluorescent chemosensors to detect cyanide anions and Cu^2+^ cations [74]. DTMC (7-(4,6-dichloro-1,3,5-triazinyl-2-amino)-4-methylcoumarin) was proposed for the chemiluminescent determination of hydrogen peroxide by the chemiluminescent method [75]. The detection limit for hydrogen peroxide is 4 × 10-8 mol/L. However, this method requires high pH values of the medium (11.4).

PFM (1-diethylaminobenzo[4,3-e]-pyran-2-hydrazone) was proposed for formaldehyde detection [76]. A year later, a more efficient fluorogenic substrate, PFM4, was proposed (Fig. 2H) [77]. PFM4 was used to successfully assess the accumulation of formaldehyde in the lysosomes of cells treated with endoplasmic reticulum stress inducers [77].

A 1995 study analyzed the effect of various enhancers on the intensity of the chemiluminescence generated in the Fe^2+^-induced peroxidation of phospholipids in egg yolk liposomes. The C-525 dye (2,3,5,6-1H,4H-tetrahydro-9-(2’-benzimidazolyl)- quinolysin-(9,9a,1-GH)) showed the most potent effect: it increased the chemiluminescence intensity more than 2,000-fold without affecting the reaction kinetics at a concentration of 4 μM [62]. The mechanism of luminescence enhancement in this case is, apparently, the energy transfer from the ketone molecule in the EES (the primary product of peroxyl radical recombination) to a fluorescent level of C-525 [60]. Meanwhile, it should be taken into account that C-525 contains a purine group, whose interaction with free radicals under certain conditions triggers an antioxidant action of the substance [78]. The specific chemiluminescence activator of the superoxide radicals 2-methyl-6-[p-methoxyphenyl]-3,7-dihydroimidazo[1,2-a]pyrazine- 3-one has a similar disadvantage [79, 80].

However, despite its structure, C-525 is quite often used as a chemiluminescence activator; e.g., when detecting lipid hydroperoxides in the lipid substrate– Fe^2+^ system [16]. Experiments in a similar system based on C-334 showed that the chemiluminescence of the system containing cytochrome c complexed with cardiolipin is due to the lipoperoxidase and quasi-lipoxygenase activity of this nanoparticle, but not to the activity of non-heme iron via the Fenton reaction [81].

The studies of the EES of coumarin derivatives should also be mentioned. Detection of photogeneration of C-314 radical cations by using nanosecond laser excitation at wavelengths > 400 nm in benzene, acetonitrile, and dichloromethane made it possible to detect the triplet EES of C-314 with maximum absorption at 550 nm and a lifetime of 90 µs in benzene, which is easily quenched by oxygen [82]. No excited state was detected in an aqueous solution; however, relatively long-lived (160 μs in air-equilibrated solutions) free C-314 radical cations with maximum absorbance at 370 nm were identified. In addition, these free C-314 radical cations are quenched by phenolic antioxidants; the rate constant for this reaction is > 109 M-1s-1 [82]. According to [82], this reaction is based on the mechanism of electron transfer between the phenolic antioxidant and C-314 radical cation with potential ionic pair formation.

A study of C-314 solvation in an aqueous solution in the presence of a surfactant [83] revealed two well-differentiated interfacial phases (water/ air). The author of the review showed that C-314, C-334, and C-525 do not dissolve in water at concentrations > 50 µM; the optimal concentration range for a coumarin derivative in the system is 20–25 µM. According to [83], surfactant addition promotes C-314 solvation. Two different positions of C-314 molecules relative to the surfactant spatial domains were revealed; they were due to large fluctuations in the surfactant concentration taking place in a small coverage area commonly called the two-dimensional gas–liquid coexistence region [83].

The mechanisms of action of various antioxidants such as β-carotene, tocopherol, rutin, and ascorbate in suppressing the lipid peroxidation triggered by free Fe^2+^ ions were studied using C-525-induced chemiluminescence [84]. The physicochemical properties of low-density plasma lipoproteins were elucidated by using the method of enhanced C-525 chemiluminescence. An increase in the amplitude of the fast luminescent flash was shown for oxidized lipoproteins in a Fe^2+^-containing solution [61]. Free-radical oxidation of cardiolipin complexed with cytochrome c was studied by detecting C-525-enhanced chemiluminescence [70].

Of special interest are the results obtained when comparing coumarin C-525 and chlorophyll-α as chemiluminescence enhancers [72]. The luminescence quantum yield was much higher in the case of C-525. A 2- to 3-fold increase in chemiluminescence accompanying the tert-butyl hydroperoxide-induced oxidation of microsomes from rat liver and peroxidation of liposomal lipids was observed. Coumarin derivatives activate chemiluminescence owing to the energy transfer from carbonyls in the triplet EES formed in the peroxide radical reaction through the Russell mechanism and dioxetane decomposition.

A very significant disadvantage of quinolizidine derivatives of coumarin should be mentioned: C-525 loses its ability to luminesce in the blood serum [55]. This is considered to be due to the binding of C-525 to serum albumins.

It has been repeatedly reported that C-314, C-334, and C-525 are fluorogenic substrates that do not react with mixture components [16, 60, 61, 62, 70]. Although these data were obtained in a non-enzymatic lipid peroxidation system [62], they were automatically projected on systems where this process is triggered by peroxidase. This was so despite the report by V.S. Sharov et al. in 1996 showing that C-525 is unsuitable for studying lipid peroxidation catalyzed by horseradish peroxidase due to the C-525 instability in this system [72].

The data indicating that quinolizidine derivatives of coumarin serve as substrates in the peroxidase reaction were confirmed in [85, 86], which showed a statistically significant decrease in the concentration of C-314, C-334, and C-525 during the peroxidase reaction catalyzed by cytochrome c complexed with cardiolipin. A decrease in the concentration of coumarin derivatives in enzymatic lipid peroxidation reduces the chemiluminescence intensity, which can lead to erroneous data interpretation: a researcher can draw a wrong conclusion about a decrease in lipid peroxidation intensity. For instance, in the case of the study of antioxidants, such a false interpretation could lead to an erroneous conclusion about an affective suppression of lipid peroxidation by the test substance. In order to avoid this trap, one should multiply the intensity values recorded by the chemiluminometer by correction factors for a decrease in the concentration of coumarin derivatives for the corresponding time points, from the beginning of the reaction when conducting an experiment on measuring the coumarin-enhanced chemiluminescence accompanying lipid peroxidation. These coefficients should be calculated using a mathematical function inverse to the decreasing function of the proportion of the concentration of coumarin derivatives, depending on the reaction time.

One should also make certain that the reaction between a coumarin derivative and peroxidase is not accompanied by luminescence. Otherwise, it is also necessary to add additional coefficients to the formula for calculating the correction factors that balance the contribution to the luminescence values recorded by the device due to the reaction between the chemiluminescence enhancer and peroxidase, not related to the luminescence accompanying lipid peroxidation.

Correction of the chemiluminescence curves obtained using the discussed correction functions allows one to return them to the form they would have had in the case of a constant concentration of the chemiluminescence enhancer in the system. Thus, it becomes possible to adequately assess enzymatic lipid peroxidation reactions in the test sample.

## Abbreviations

DTMC: 7-(4,6-dichloro-1,3,5-triazinylamino)-4-methylcoumarin
C-314: coumarin-314 - quinolizidine[5,6,7-gh]3-ethoxycarbonylcoumarin
C-334: coumarin-334 - quinolizidine[5,6,7-gh]3-acetylcoumarin
C-525: coumarin-525 - quinolizidine[5,6,7-gh]3,2’-benzimidazolylcoumarin
EES: electronically excited state
ROS: reactive oxygen species
EEE: electronic excitation energy.
